# Modeling the Influence of *CYP2C9* and *ABCB1* Gene Polymorphisms on the Pharmacokinetics and Pharmacodynamics of Losartan

**DOI:** 10.3390/pharmaceutics17070935

**Published:** 2025-07-20

**Authors:** Dmitry Babaev, Elena Kutumova, Fedor Kolpakov

**Affiliations:** 1 Department of Computational Biology, Sirius University of Science and Technology, 354340 Sirius, Russia; 2 Laboratory of Bioinformatics, Federal Research Center for Information and Computational Technologies, 630090 Novosibirsk, Russia

**Keywords:** arterial hypertension, losartan, pharmacokinetics, pharmacodynamics, *ABCB1*, *CYP2C9*, mathematical modeling, personalized medicine, BioUML

## Abstract

**Background/Objectives:** Hypertension is a pathological condition characterized by elevated systolic and/or diastolic blood pressure. A range of pharmacotherapeutic agents are available to treat this condition and prevent complications, including the angiotensin II AT1-receptor blocker losartan. Following oral administration, losartan is exposed to a variety of enzymes that facilitate its metabolism or transportation. The structural characteristics of the genes that encode the enzymes may potentially impact the pharmacokinetics and pharmacodynamics of losartan, thereby modulating its effects on the treatment process. **Methods**: In this study, a computational model of losartan pharmacokinetics was developed, taking into account the influence of different alleles of the *CYP2C9* gene, which plays a pivotal role in losartan metabolism, and the *ABCB1* gene, which is responsible for losartan transport. **Results**: Alterations in the modeled activities of the enzymes encoded by *CYP2C9* and *ABCB1* result in changes in the losartan and its metabolite profiles that are consistent with known experimental data in real patients with different *CYP2C9* and *ABCB1* genotypes. **Conclusions**: The findings of the modeling can potentially be used to personalize drug therapy for arterial hypertension.

## 1. Introduction

Members of the adenosine triphosphate binding cassette (ABC) transporter superfamily are predominantly membrane-bound enzymes that catalyze ATP-dependent translocation of a wide variety of substrates across cellular membranes [1,2]. Members of this superfamily are organized into seven families (A–G) on the basis of their sequence similarity.

The ABCB1 (ATP-binding cassette superfamily, subfamily B, member 1) protein, also known as MDR1 (multidrug resistance 1) or P-glycoprotein, was first identified in cultured cancer cells. Subsequent studies demonstrated that the overexpression of ABCB1 in these cells resulted in their cross-resistance to many drugs [3,4]. The *ABCB1* gene is located on the minus strand of chromosome 7q21.12, spanning 32 exons (https://www.ncbi.nlm.nih.gov/gene/5243, accessed on 5 June 2025) and encoding a protein of 1280 amino acid residues (https://www.ncbi.nlm.nih.gov/protein/P08183.3, accessed on 5 June 2025), with a molecular weight of 170 kDa [5].

ABCB1 is also expressed in various types of normal organs and tissues, where it restricts the entry of different substances. For example, this protein is found in the brain [6,7], gastrointestinal tract [8], adrenal glands, kidneys, liver, and testes [9,10,11].

*ABCB1* is a highly polymorphic gene, with more than 8000 single nucleotide polymorphisms (SNPs) having been reported [12]. Among these substitutions, three are the most common in the coding region: (1) a cytosine to thymine transversion at nucleotide 1236 (C1236T) [13], (2) a guanine to thymine or adenine substitution at nucleotide 2677 (G2677T/A) [14], and (3) a cytosine to thymine transversion at nucleotide 3435 (C3435T) [13] (Table 1).

**Figure 1:**
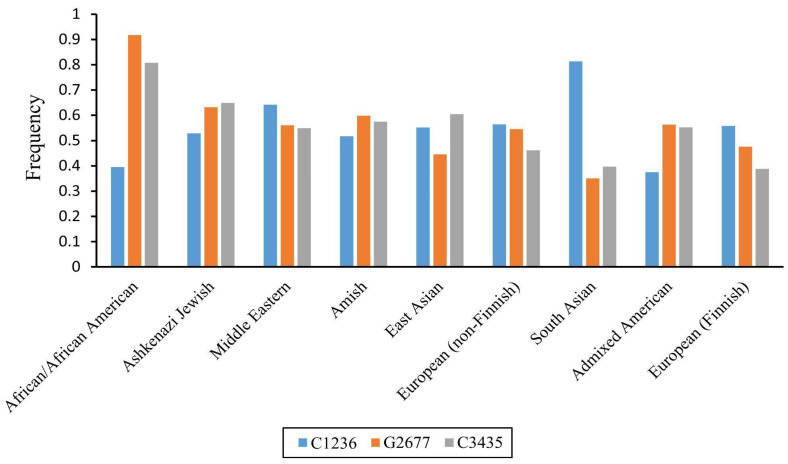
*ABCB1* allele frequencies (data from gnomAD v4.1.0 https://gnomad.broadinstitute.org/, accessed on 5 June 2025).

The frequencies of all the aforementioned polymorphisms vary significantly among different ethnic groups (Figure 1). Furthermore, studies have demonstrated that these three SNPs are in strong linkage disequilibrium, forming the two most common haplotypes: ABCB*1 (C1236/G2677/C3435) and ABCB1*13 (T1236/T2677/T3435) [15,16,17,18,19,20]. It was also shown that the ABCB1*13 haplotype is associated with reduced protein expression in the duodenum compared to the ABCB1*1 haplotype [13]. Subsequent analyses revealed that this phenomenon is associated with diminished stability of the mRNA expressed from the ABCB1*13 allele, which is a consequence of the C3435T substitution [21].

*ABCB1* is a broad-spectrum enzyme that can transport a variety of drugs, including losartan, a selective angiotensin II AT1-receptor antagonist used to treat arterial hypertension and heart failure [22,23]. Within the organism, losartan is converted to carboxylosartan (E-3174) via a carbonyl intermediate (E-3179), primarily by CYP2C9 (cytochrome P450 superfamily, family 2, subfamily C, member 9) [24,25,26]. E-3174 is thought to be responsible for the main pharmacological effect, as it has 10–40 times greater AT1-receptor blocking activity than losartan and possesses a longer half-life [27].

*CYP2C9* is also a polymorphic gene, with the most common alleles being CYP2C9*1 (wild-type), CYP2C9*2, and CYP2C9*3 [28,29]. The CYP2C9*2 variant refers to a gene with a cytosine to thymine transversion at nucleotide 430 (C430T), leading to an arginine to cysteine replacement at amino acid residue 144 (Arg144Cys) [30,31]. An adenine to cytosine transversion at nucleotide 1075 (A1075C) encodes for an isoleucine to leucine substitution at amino acid residue 359 (Ile359Leu), producing the CYP2C9*3 variant allele [32] (Table 2). It was shown that the presence of the CYP2C9*2 or CYP2C9*3 alleles is associated with decreased enzyme activity in both in vitro [29] and in vivo [33] settings; however, the most substantial reductions were observed with the CYP2C9*3 variant. Furthermore, studies have indicated that this allele is associated with adverse reactions to drugs that are substrates of CYP2C9 [34,35].

This work is a continuation of our research into modeling the effect of genetic factors on the pharmacokinetics and pharmacodynamics of antihypertensive drugs [36,37,38]. Previously, we developed a model describing the pharmacokinetics of losartan in relation to different *CYP2C9* alleles [36,37]. Here we extended this model by adding the influence of different *ABCB1* variant alleles on the pharmacokinetics of losartan and E-3174. Along with this, we conducted in silico studies to examine the blood pressure response to losartan therapy depending on the *ABCB1* genotype using the previously created mechanistic cardiorenal model [39,40]. The results of this work can be used for comprehensive analysis of the development and treatment of arterial hypertension.

## 2. Materials and Methods

### 2.1. Mathematical Model of Losartan Metabolism with ABCB1 Influence

**Figure 2:**
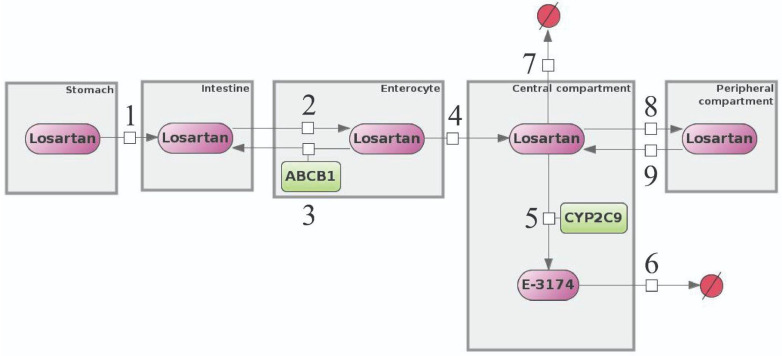
A model of losartan metabolism with a new compartment, “Enterocyte”. Numbers 1 through 9 denote reactions, compartments are shown as gray rectangles, purple ovals represent chemical compounds, excreted substances are shown as crossed-out red circles, and green rectangles denote enzymes.

We have modified our previous model [36,37] by adding a new compartment, “Enterocyte”, that illustrates the enterocyte cells of the intestine (Figure 2).

The model includes five distinct compartments: the stomach, intestine, enterocyte, central, and peripheral compartments. These compartments contain two substances, losartan and E-3174, which are interconnected through first-order reaction equations (numbers 1 through 9 in Figure 2). It is noteworthy that reaction 5, which is responsible for the CYP2C9 activity, is described by a first-order delayed differential equation. A detailed description of all reaction equations and variables is given in Supplementary Tables S1 and S2, respectively. The model also includes a discrete event to describe the delayed action of CYP2C9, an initial assignment to specify oral administration of losartan and its entry into the stomach, and algebraic equations. These equations are used to calculate the rate constants of the model reactions and the concentrations of losartan and E-3174 at each time point during the simulation. A full list of the mathematical tools and variables used can be found in Supplementary Tables S3 and S4, respectively.

### 2.2. Nomenclature of Genotypes

The nomenclature employed to describe *CYP2C9* genotypes includes labels such as CYP2C9*1/CYP2C9*2, where CYP2C9*1 is the first allele of the *CYP2C9* gene and CYP2C9*2 is the second allele of the same gene. In total, we examined 3 *CYP2C9* alleles: CYP2C9*1 (wild-type), CYP2C9*2 (C430T), and CYP2C9*3 (A1075C).

In the case of the *ABCB1* gene, we investigated several groups of SNPs, i.e., haplotypes: CC/GG/CC, TT/TT/TT, GG/CC, GT/CT, and TT/TT. The first and second letters in these labels denote nucleotides at position 1236 in the first and second alleles of the *ABCB1* gene, respectively; the third and the fourth letters, nucleotides at position 2677; and the fifth and the sixth letters, nucleotides at position 3435. In 4-digit labels the first and the second letters represent nucleotides at position 2677, while the third and the fourth letters refer to nucleotides at position 3435.

### 2.3. Pharmacokinetic Analysis

The pharmacokinetic parameters used for the analysis are presented in Table 3.

*C*_max_ and *t*_max_ were derived from the model simulation results. AUC_0–∞_ was calculated using the following differential equation:

$$\frac{d\left({\mathrm{AUC}}_{0-\infty }\right)}{dt}=C,$$

where *C* is the concentration of the compound.

The calculation of AUC_x–y_ was performed in three steps:

1. AUC from 0 to the lower boundary (x) was derived:
  $$\frac{d({\mathrm{AUC}}_{0-x})}{dt}=\left\{\begin{array}{l} 0,\;\mathrm{if}\;t\;>\;x,\\ C,\;\mathrm{otherwise}. \end{array}\right.,$$
  where *C* is the concentration of the substance;
2. AUC from 0 to the upper boundary (y) was derived:
  $$\frac{d({\mathrm{AUC}}_{0-y})}{dt}=\left\{\begin{array}{l} 0,\;\mathrm{if}\;t\;>\;y,\\ C,\;\mathrm{otherwise}. \end{array}\right.,$$
  where *C* is the concentration of the substance;
3. AUC from x to y was calculated as the difference between AUC_0–y_ and AUC_0–x_:
  $${\mathrm{AUC}}_{x-y}={\mathrm{AUC}}_{0-y}-{\mathrm{AUC}}_{0-x}.$$

The calculation of *t*_1/2_ was performed in two steps:

1. A linear regression line was derived for the semilogarithmic plot of plasma concentration versus time using the 6, 8, and 10 h time points, as in the article from which data were extracted for model validation [41].
2. *t*_1/2_ was calculated:
  $$t_{1/2}=\frac{\left(t_{2}-t_{1}\right)\times \ln\left(2\right)}{\ln\left(\frac{C_{1}}{C_{2}}\right)},$$
  where *C*_1_ and *C*_2_ are the concentrations of the substance at times *t*_1_ and *t*_2_, respectively, according to the derived linear regression line.

The calculation of CL/F was performed using a piecewise function, since the concentration of losartan in the initial numerical steps of the model simulation is zero:

$$CL/F=\left\{\begin{array}{l} 0,\;\mathrm{if}\;C\_p=0,\\ \frac{oral_{dose}\times 10^{6}}{{\mathrm{AUC}}_{0-\infty ,\;\mathrm{losartan}}\times 461.01},\;\mathrm{otherwise}. \end{array}\right.,$$

where 10^6^ is the conversion coefficient from milligrams to nanograms and 461.01 is the molecular weight of losartan potassium (g/mol).

### 2.4. Virtual Population for Mathematical Cardiorenal Model

To assess the antihypertensive effect of losartan in a population of virtual patients with different *ABCB1* genotypes, we used the computational model of the human cardiovascular and renal systems that, in particular, simulates the pharmacological effects of losartan on cardiovascular and renal parameters [39,40]. This model is discrete–continuous and consists of a system of ordinary differential equations with several discrete events corresponding to instantaneous changes in the modeled dynamics (e.g., the transition from systole to diastole). This model is available in the BioModels database [42] with ID MODEL2202160001 (https://www.ebi.ac.uk/biomodels/MODEL2202160001, accessed on 5 June 2025).

A virtual patient is defined as an equilibrium state of a cardiorenal model within specified physiological constraints. A virtual population is a set of unique virtual patients.

### 2.5. Modeling the Impact of ABCB1 Genetic Variants on Losartan Treatment Response

To simulate the treatment of virtual patients with losartan, we used the following equation from the cardiorenal model [40], which reduces the rate of angiotensin II binding to AT1-receptors:

$$ARB=k_{block}\times {\mathrm{Losartan}}_{treatment},$$

where *ARB* is the AT1-receptor blocking activity of the drug, *Losartan_treatment_* is a discrete parameter that can take values of 0 (no treatment) or 1 (treatment course), and *k_block_* is a constant.

The value of the parameter *k_block_* is entered into the cardiorenal model from the losartan metabolism model, and it was estimated using the following considerations. Losartan 25 mg orally once daily has not been shown to produce clinically significant reductions in blood pressure compared with placebo [43]. A similar effect in the cardiorenal model is given by *k_block_* = 0.1. For daily oral doses of 50 and 100 mg losartan, *k_block_* values were previously estimated [40] to be 0.886 and 0.954, respectively. Using these doses in the losartan metabolism model with parameters fitted to the *ABCB1* GG/CC genotype (the most active haplotype, as defined by [13,22,44]), we calculated the corresponding AUC_0–∞, E-3174_ values as E-3174 is thought to be responsible for the main pharmacological effect of losartan [27].

We then used the following E-max model [45] to describe the dependence between *k_block_* and AUC_0–∞, E-3174_:

(1) $$k_{block}=\frac{E_{\max}\times {{\mathrm{AUC}}_{0\text{–}\infty ,E\text{-}3174}}^{\alpha }}{{ED_{50}}^{\alpha }+{{\mathrm{AUC}}_{0\text{–}\infty ,E\text{-}3174}}^{\alpha }}.$$

Using the three dependence points between *k_block_* and AUC_0–∞, E-3174_ for the GG/CC genotype, we fitted the values of the E-max model (1) parameters E_max_, ED_50_, and α to ensure that the E-max model function approximated the experimental points with optimal accuracy. The resulting curve is presented in Supplementary Figure S1.

The coefficients of the fitted E-max model are presented in Table 4.

Finally, the AUC_0–∞, E-3174_ values were determined for each *ABCB1* genotype (GG/CC, GT/CT, and TT/TT) at two losartan doses (50 mg and 100 mg) using our losartan metabolism model. These values were then applied to the E-max model (1) with the fitted coefficients (Table 4), yielding the *k_block_* value for each of the six cases (Supplementary Table S5).

### 2.6. Parameter Estimation

To solve the inverse problem of identifying model parameters based on experimentally measured variables, three nonlinear optimization methods were tested: SRES (Stochastic Ranking Evolutionary Strategy) [46], MOPSO (Multi-Objective Particle Swarm Optimization) [47], and MOCell (Multi-objective cellular genetic algorithm) [48]. Since MOPSO showed the best solution for the problem under consideration, the results obtained with this method are presented.

### 2.7. Parameter Identifiability

After estimating the model parameters based on experimental data, it is important to understand how accurately these parameters have been estimated in terms of the quantity and quality of the data. This understanding is necessary for further investigation of model predictions and can be provided by analyzing the parameters for identifiability [49,50]. To study the sensitivity of the objective function to changes in a fitting parameter, we exclude it from the optimization process with a fixed value that gradually increases and then decreases compared to the optimal solution. In this way, we determine the influence of this parameter on the value of the objective function (i.e., the quality of the experimental data approximation). If the shift of the parameter in any direction along the numerical axis leads to a significant increase in the objective function, then it is identifiable. If a significant increase in the objective function occurs when moving in only one direction, then the parameter is partially identifiable. Otherwise, it is impossible to determine the parameter based on the available experimental data, that is, it is unidentifiable.

### 2.8. Sensitivity Analysis

To examine the sensitivity of the simulation results to perturbations in the model parameters, we calculated their relative coefficients (*SS*) [51]:

$$SS=\frac{C_{SS}\left(\alpha +\Delta \alpha \right)-C_{SS}\left(\alpha \right)}{\Delta \alpha }\times \frac{\alpha }{C_{SS}\left(\alpha \right)},$$

where α is the initial value of the parameter, Δα (10^−6^ in our case) is the perturbation value, and *C_SS_* (α) and *C_SS_* (α + Δα) are the simulated values of the tested variable with the initial and altered values of the parameter, respectively.

### 2.9. Digitizing of Plots

To train the losartan metabolism model, we used experimental plasma concentrations of the drug and its active metabolite following an oral dose of 50 mg losartan potassium in individuals with different *CYP2C9* and *ABCB1* genotypes: CYP2C9*1/CYP2C9*1; CYP2C9*3/CYP2C9*3 [52] (Supplementary Table S6); and GG/CC, GT/CT, TT/TT [41] (Supplementary Table S7). We also extracted data on the systolic and diastolic blood pressure response to losartan treatment (daily oral dose of 100 mg for 6 weeks) for *ABCB1* genotypes CC/GG/CC and TT/TT/TT [53] (Supplementary Table S8).

### 2.10. Statistical Analysis

Differences in the pharmacokinetic parameters of losartan and E-3174 among the three *ABCB1* genotypes (GG/CC, GT/CT, and TT/TT) were assessed using a Kruskal–Wallis one-way ANOVA on ranks. This approach was employed because the data for at least one of the comparison groups did not follow a normal distribution (Shapiro–Wilk test, *p* < 0.05) or dispersions between the comparison groups were unequal (equal variance test, *p* < 0.05). Pairwise comparisons were performed using the *t*-test (Shapiro–Wilk test, *p* > 0.05; equal variance test, *p* > 0.05) or the Mann–Whitney rank sum test (Shapiro–Wilk test, *p* < 0.05 or equal variance test, *p* < 0.05). In both cases, the Bonferroni cutoff for significance was used because there were 3 comparison groups. *p* < 0.05 was considered statistically significant.

### 2.11. Software

To develop and analyze the model, we used the BioUML (http://www.biouml.org/, accessed 5 June 2025) software (version 2025.2), a Java-based integrated environment for the modeling of different biological systems [54,55]. The model was created using the SBML format for technical representation [56] and the SBGN format for visualization [57].

To simulate the models, we used a version of the CVODE solver [58] ported to Java and adapted to the BioUML software interface.

To digitize the data from the figures in the original study, we used WebPlotDigitizer (https://apps.automeris.io/wpd4/, accessed 5 June 2025) software (version 4.8).

Statistical analysis was performed using the SigmaPlot 12^®^ program, version 12.5 (Systat Software Inc., San Jose, CA, USA).

## 3. Results

### 3.1. Model Developing

In the current study, we added a new compartment, “Enterocyte”, to the initial model describing the pharmacokinetics of losartan in relation to different *CYP2C9* alleles [36,37] to take into account the influence of the ABCB1, since it has been shown that this enzyme is located on the wall of the small intestine, where it transports losartan back into the intestinal lumen [8,22]. This novel compartment lies between the “Intestine” and the central compartment, and they are interconnected through three first-order reactions of losartan transport: (1) from the “Intestine” to the “Enterocyte”, (2) from the “Enterocyte” to the “Intestine” (action of the ABCB1), and (3) from the “Enterocyte” to the central compartment (see “Mathematical model of losartan metabolism with ABCB1 influence” in Section 2).

### 3.2. Analysis of Experimental Data for Model Validation

To validate our model, it was necessary to obtain experimental measurements of plasma concentrations of losartan and E-3174 in individuals with different *CYP2C9* and *ABCB1* genotypes after oral losartan administration. However, we could not find any clinical trials that examined both genes in conjunction. Consequently, we decided to use data from different studies for each of these genes.

**Figure 3:**
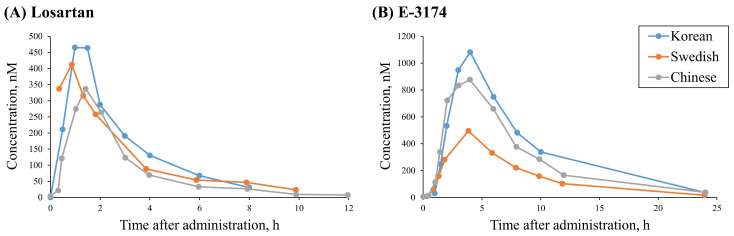
Comparisons between the profiles of losartan (**A**) and E-3174 (**B**) after a single 50 mg oral dose of losartan potassium in CYP2C9*1/CYP2C9*1 individuals of different ethnicities: Korean [52], Swedish [33], and Chinese [59].

In the case of *CYP2C9*, we compared the data from three studies conducted on patients of different ethnicities: Korean [52], Swedish [33], and Chinese [59]. Despite the fact that the same oral dose of losartan was used in all these trials (50 mg), the authors obtained similar concentration–time profiles of losartan (Figure 3A), but significantly divergent data for E-3174 (Figure 3B) for the CYP2C9*1/CYP2C9*1 genotype. We assumed that the observed discrepancy was attributable to the other factors that may vary between different ethnic groups, such as lifestyle, diet, climate, genotypes of other genes, etc.

In the case of *ABCB1*, we found only one study [41] comparing the concentration–time profiles of losartan and E-3174 in Korean subjects with different genotypes (GG/CC, GT/CT, and TT/TT).

Thus, we decided to use data obtained from Korean individuals for both the *CYP2C9* [52] and *ABCB1* [41] genes to minimize interethnic variability (Figure 3B).

Then, it was necessary to make an assumption about the “missing” gene, given that the study on patients with different *CYP2C9* genotypes [52] lacked the information regarding *ABCB1*, and, conversely, in individuals with known *ABCB1* genotype [41], the *CYP2C9* gene was not examined.

**Figure 4:**
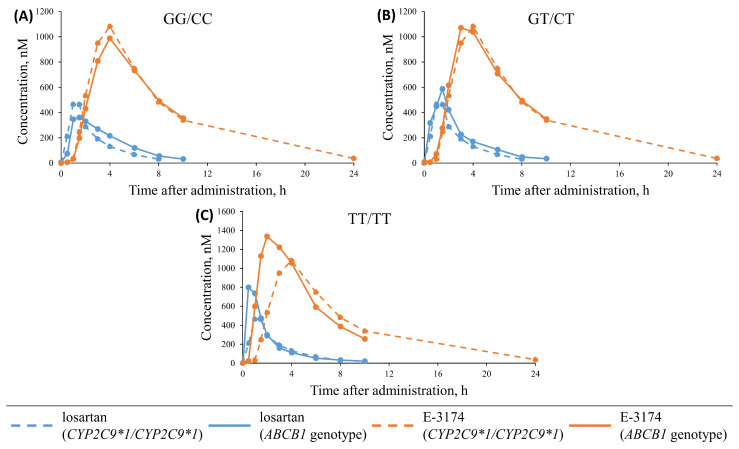
Comparison of the concentration–time curves for losartan (blue) and E-3174 (orange) between CYP2C9*1/CYP2C9*1 [52] (dashed lines) and different *ABCB1* genotypes: GG/CC (**A**), GT/CT (**B**), and TT/TT (**C**) [41] (solid lines).

For the “missing” *CYP2C9* genotype, we assumed that all individuals were CYP2C9*1/CYP2C9*1, as this allele is the most common in the Korean population (95%) [52]. To reveal the “missing” *ABCB1* genotype, the same approach was not applicable, because the frequencies of SNPs in this gene are approximately 0.5 (Figure 1, “East Asian”), and it was not possible to consider an exact genotype. Therefore, we compared losartan and E-3174 concentration–time curves between CYP2C9*1/CYP2C9*1 and all *ABCB1* genotypes investigated (GG/CC, GT/CT, and TT/TT) [41] to find out which of these corresponded most closely to the wild-type genotype of *CYP2C9* (Figure 4).

To assess the difference between the curves, we used the distance formula:

$$D=\sqrt{\sum_{i=1}^{j}{\left(c_{i}^{\mathrm{CYP}2C9*1/\mathrm{CYP}2C9*1}-c_{i}^{\mathrm{ABCB}1}\right)}^{2}},$$

where *D* is the deviation between two experimental datasets, *j* is the number of time points that are common to both datasets, and c_i_^CYP2C9*1/CYP2C9*1^ and *c_i_^ABCB1^* are the concentrations of losartan or E-3174 at time point *i* for the CYP2C9*1/CYP2C9*1 genotype or one of the *ABCB1* genotypes, respectively.

As can be seen in Table 5, the greatest agreement was observed for the GG/CC and GT/CT genotypes. In contrast, the TT/TT genotype significantly deviated from the concentration–time profiles of the CYP2C9*1/CYP2C9*1 genotype. To determine whether the GG/CC or GT/CT variant is more prevalent in patients with an unknown *ABCB1* genotype [52], we analyzed an additional experimental study involving Koreans [60]. The genotyping results yielded the following distribution: 51 patients (40.5%) had the GT/CT genotype, 23 patients (18.3%) were GG/CC, 12 patients (9.5%) were TT/TT, and the remaining 40 individuals (31.7%) had other uncommon genotypes (GA/CC, AA/CC, AT/CC, GT/CC, TT/TC, TG/CT, and GG/TT). As can be seen, the GT/CT genotype was the most frequent in this cohort of patients. Therefore, we assumed that all individuals with an unknown *ABCB1* genotype [52] were carriers of GT/CT.

### 3.3. Validation of the Model

The validation of the model was carried out in two steps. Firstly, we determined the values of the parameter *k_m*, which characterizes the rate of conversion of losartan to E-3174, i.e., the activity of CYP2C9 (Figure 2, reaction 5). Using the experimental data for the *CYP2C9* genotypes [52], we estimated the unique value of this parameter for each of the two homozygous genotypes:

$$k\_m\left(CYP2C9*1/CYP2C9*1\right)=\;2.817{\;h}^{-1},$$

$$k\_m\left(CYP2C9*3/CYP2C9*3\right)=0.039\;h^{-1}.$$

During this initial stage, all other parameters of the model were estimated using general values for both CYP2C9*1/CYP2C9*1 and CYP2C9*3/CYP2C9*3 genotypes, since we assumed that patients only differed in their *CYP2C9* genotype.

The CYP2C9*2 allele was excluded from the analysis due to its rarity within the Korean population, as confirmed by previous studies [52,61,62,63].

Using then the fixed *k_m* values obtained in the previous step for the experimental data not only for *CYP2C9*, but also for *ABCB1* genotypes [41] (CYP2C9*1/CYP2C9*1, CYP2C9*3/CYP2C9*3, GG/CC, GT/CT, and TT/TT, in total), we defined the values of all other model parameters, including the unique values of the *k_ent_int*, which characterizes the activity of the ABCB1 (Figure 2, reaction 3), for three newly added genotypes:

$$k\_ent\_int\left(\mathrm{GG}/\mathrm{CC}\right)=151.485\;h^{-1},$$

$$k\_ent\_int\left(\mathrm{GT}/\mathrm{CT}\right)=101.800\;h^{-1},$$

$$k\_ent\_int\left(\mathrm{TT}/\mathrm{TT}\right)=1.431\times 10^{-12}\;h^{-1}.$$

For this final estimation step, we used the initial values of the model parameters obtained in the previous step.

The final optimized values of all other parameters are listed in Supplementary Table S9.

**Figure 5:**
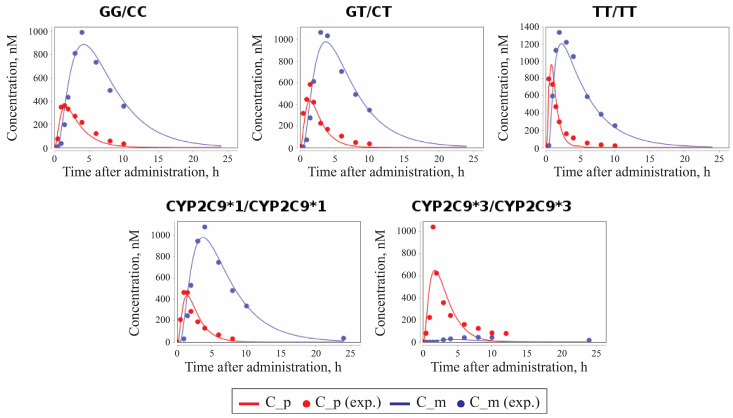
Model prediction of losartan and E-3174 plasma concentrations for two *CYP2C9* and three *ABCB1* genotypes after a single 50 mg oral dose of losartan potassium. C_p, predicted profile of losartan; C_m, predicted profile of E-3174; C_p (exp.), experimental time-course of losartan; C_m (exp.), experimental time-course of E-3174. In experimental works, 13 patients were included in GG/CC group, 12 patients in the GT/CT group, 13 patients in the TT/TT group [41], 12 patients in the CYP2C9*1/CYP2C9*1 group, and only 1 patient in the CYP2C9*3/CYP2C9*3 group [52].

The time-dependent concentrations of losartan and E-3174 for two *CYP2C9* and three *ABCB1* genotypes predicted by the model after parameter redefinition, as well as the experimentally obtained data for individuals with the same genotypes [41,52], are shown in Figure 5.

After optimizing the model parameters, we checked them for identifiability to ensure that the resulting solution was unique. As can be seen from Supplementary Figure S2, all model parameters are identifiable.

### 3.4. Comparison of Simulated and Experimental Pharmacokinetic Parameters

Following the validation of the model, we compared the pharmacokinetic parameter values from the clinical studies [41,52] with those predicted by the model.

For the CYP2C9*1/CYP2C9*1 genotype, the simulated values of the following pharmacokinetic parameters are outside the mean ± *SD* experimental range: *t*_1/2, losartan_, *C*_max, E-3174_, and *t*_1/2, E-3174_. Meanwhile, CL/F falls within this range, but is slightly lower than the 95% confidence interval (80.182 vs. 82–106 L/h). The group of patients with the CYP2C9*3/CYP2C9*3 genotype included only one individual [52], precluding the possibility of conducting a statistical analysis. Consequently, a comparison of simulated and clinically obtained values could not be made in this case (Table 6).

The comparison for the *ABCB1* genotypes is shown in Table 7. The simulated values of the *t*_1/2, losartan_ (all genotypes), *t*_1/2, E-3174_ (GT/CT and TT/TT), and *C*_max, losartan_ (GT/CT) are outside the mean ± *SD* experimental range, while all other parameters are in the same range. We also compared simulated and experimental AUC_losartan+E-3174_ for different time periods (Supplementary Table S10).

**Figure 6:**
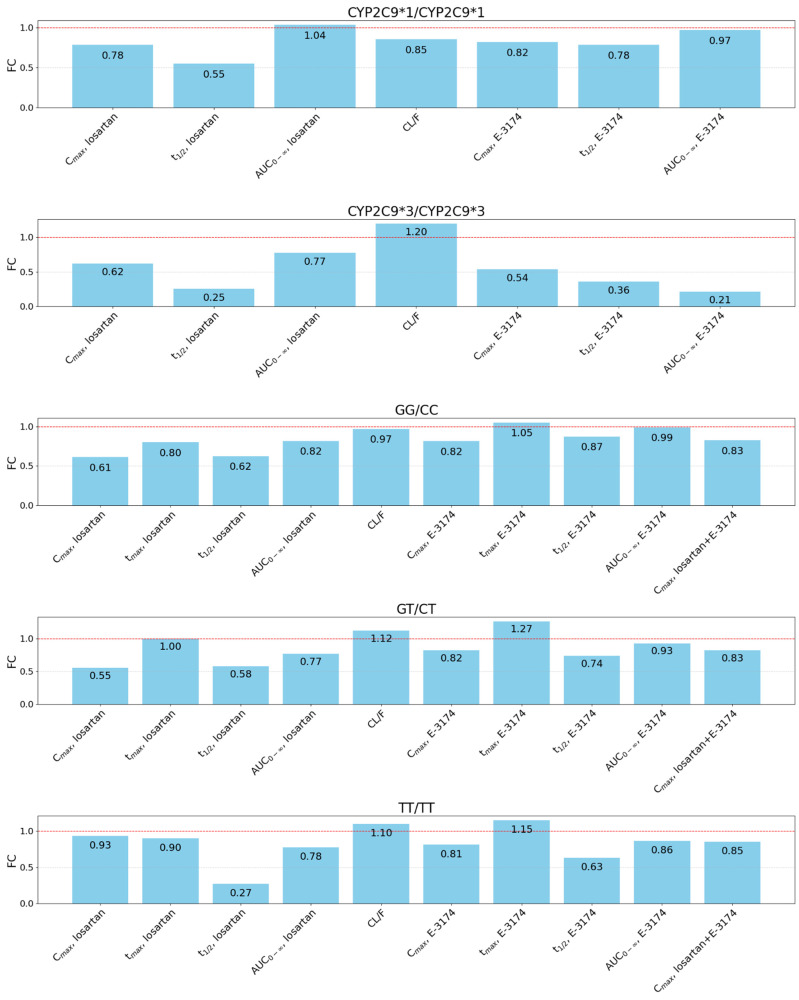
Plots demonstrating the ratio of predicted to experimental values or fold change (FC) for all pharmacokinetic parameters for the following genotypes: CYP2C9*1/CYP2C9*1, CYP2C9*3/CYP2C9*3, GG/CC, GT/CT, and TT/TT. *C*_max_, maximum plasma concentration; *t*_max_, time at which *C*_max_ occurred; *t*_1/2_, terminal elimination half-life; AUC_0–∞_, area under the concentration–time curve from zero to infinity; CL/F, apparent oral clearance of losartan. Red dashed lines indicate the level of FC = 1.

Plots demonstrating the ratio of predicted to experimental values for all pharmacokinetic parameters for each genotype can be found in Figure 6.

**Figure 7:**
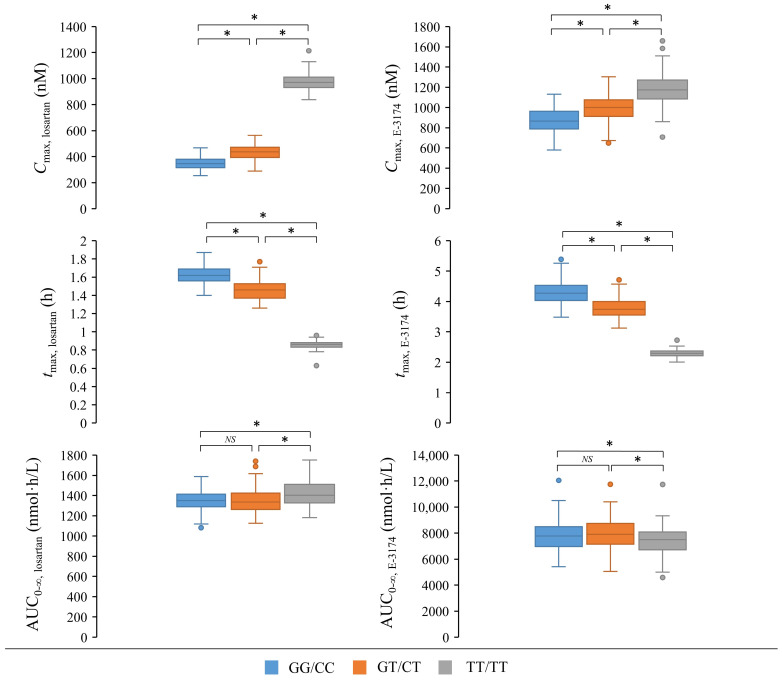
Comparison of the *C*_max_ (maximum plasma concentration), *t*_max_ (time at which *C*_max_ occurred), and AUC_0–∞_ (area under the concentration–time curve from zero to infinity) of losartan and E-3174 for simulated *ABCB1* genotypes. Box plots show medians, interquartile ranges, maximum, minimum, and outliers. *, *p*-value < 0.0167 (0.05/number of *ABCB1* genotypes, Bonferroni cutoff for significance). *NS*, *p*-value > 0.0167 (0.05/number of *ABCB1* genotypes, Bonferroni cutoff for significance). A total of 100 values were used to create each boxplot. The most significant differences were observed between TT/TT and other genotypes (GG/CC+GT/CT) for *C*_max, losartan_, *t*_max, losartan_, and *t*_max, E-3174_.

Next, we decided to compare the simulated values of key pharmacokinetic parameters for different *ABCB1* genotypes with each other. Firstly, we modeled the between-subject variability of losartan and E-3174 plasma curves. We assumed that the optimized values of the model parameters (Supplementary Table S9) corresponded to the median values, while *SD* was 10% of these values (Supplementary Table S11). Then, using a derived normal distribution, we randomly selected 100 values for each model parameter for each *ABCB1* genotype (300 values in total). Supplementary Figure S3 shows the distributions of the global model parameters, which are consistent across all genotypes and therefore have a single value for each genotype. In contrast, Supplementary Figure S4 demonstrates the separate distribution plots of the *k_ent_int* local parameter, which characterizes ABCB1 activity and consequently exhibits different values for various *ABCB1* genotypes. Finally, we compared the values of *C*_max_, *t*_max_, and AUC_0–∞_ for losartan and E-3174 across the generated parameter sets (Figure 7).

### 3.5. Sensitivity Analysis

**Figure 8:**
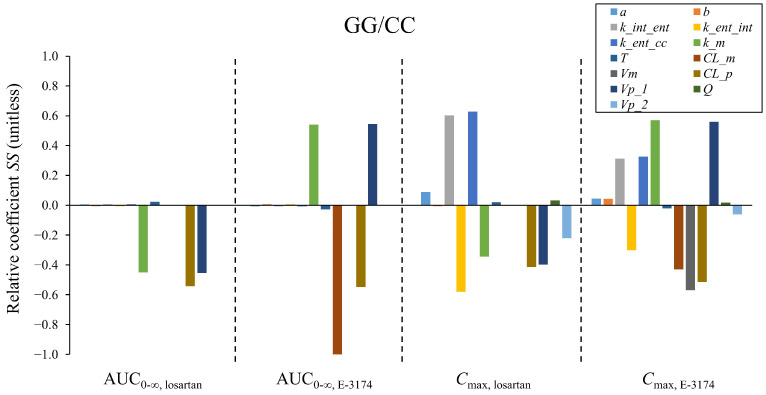
Relative coefficients *SS* of the model parameters for the GG/CC genotype. *a*—the amplitude of the sinusoidal equation, which describes open–close cycles of the gastric pyloric valve (h^−1^); *b*—the period of the sinusoidal equation, which describes open–close cycles of the gastric pyloric valve (h); *k_int_ent*—rate constant of losartan absorption from the intestinal lumen into enterocytes (h^−1^); *k_ent_int*—rate constant of reverse transport of losartan from enterocytes to the intestinal lumen by ABCB1 (h^−1^); *k_ent_cc*—rate constant of losartan absorption from enterocytes into the blood (h^−1^); *k_m*—rate constant of the conversion of losartan to E-3174 by CYP2C9 (h^−1^); *T*—time delay in the conversion of losartan to E-3174 (h); *CL_m*—apparent clearance of E-3174 (L/h); *Vm*—apparent volume of distribution of E-3174 in the blood (L); *CL_p*—apparent clearance of losartan (L/h); *Vp_1*—apparent volume of distribution of losartan in the blood (L); *Q*—apparent clearance of losartan transfer between the blood and other organs and tissues (L/h); *Vp_2*—apparent volume of distribution of losartan in other organs and tissues (L). AUC_0–∞_—area under the concentration–time curve from zero to infinity (nmol·h/L); *C*_max_—maximum plasma concentration (nM).

To examine the impact of perturbations in model parameters on simulation results, we performed a sensitivity analysis for a single *ABCB1* genotype. For illustrative purposes, we chose the GG/CC genotype (Figure 8).

The most sensitive parameters for AUC_0–∞, losartan_ are *k_m* (rate constant of the conversion of losartan to E-3174 by CYP2C9), *CL_p* (apparent clearance of losartan), and *Vp_1* (apparent volume of distribution of losartan in the blood). *k_m* and *CL_p* are responsible for the conversion and elimination of losartan, respectively; therefore, their increase leads to a decline in AUC_0–∞, losartan_ (Figure 8). *Vp_1* is a complex parameter, which is involved in several reactions of the model (Supplementary Tables S3 and S4), and, consequently, it is difficult to accurately assess the influence of its perturbation on the model simulation.

In the context of AUC_0–∞, E-3174_, the most significant parameters are *k_m*, *CL_m* (apparent clearance of E-3174), *CL_p*, and *Vp_1*. *k_m* has the opposite effect than in the case of AUC_0–∞, losartan_, while *CL_m* and *CL_p* have negative coefficients, as their increment causes decreases in E-3174 and losartan concentrations in the central compartment, respectively.

The most sensitive parameters for *C*_max, losartan_ are *k_int_ent* (rate constant of losartan absorption from the intestinal lumen into enterocytes), *k_ent_int* (rate constant of reverse transport of losartan from enterocytes to the intestinal lumen by ABCB1), and *k_ent_cc* (rate constant of losartan absorption from enterocytes into the blood). These parameters are involved in losartan transport reactions in the enterocyte (Figure 2, Supplementary Tables S1 and S2) and determine the amount of losartan in the central compartment and, consequently, its maximum concentration.

For *C*_max, E-3174_ the most essential parameters are *k_m*, *Vm* (apparent volume of distribution of E-3174 in the blood), and *Vp_1*. *k_m* has a positive coefficient as in the case of AUC_0–∞, E-3174_, while *Vm*, like *Vp_1*, is involved in several model reactions simultaneously (Supplementary Tables S3 and S4).

We also calculated the relative coefficients of the model parameters for different *ABCB1* genotypes (Supplementary Table S12).

### 3.6. Simulation of Losartan Antihypertensive Therapy

To assess the antihypertensive effect of losartan therapy in individuals with different *ABCB1* genotypes, we used a previously developed cardiorenal model [39,40], which, in particular, reproduces the pharmacological action of losartan. The study examined 100 virtual patients with arterial hypertension generated for it earlier [40]. The distribution of their physiological characteristics is presented in Supplementary Figure S5. To test how virtual patients with different allelic variants of *ABCB1* would respond to losartan treatment, we estimated the values of the *k_block_* parameter, representing the AT1-receptor blocking activity of the drug, for various *ABCB1* genotypes, as well as for 50 and 100 mg oral doses of losartan potassium as described in the “Materials and Methods” subchapter “Modeling the impact of *ABCB1* genetic variants on losartan treatment response”. Each of the 100 virtual patients was simulated with each of the *k_block_* values, i.e., the difference between the groups for each genotype consisted only in ABCB1 activity.

**Figure 9:**
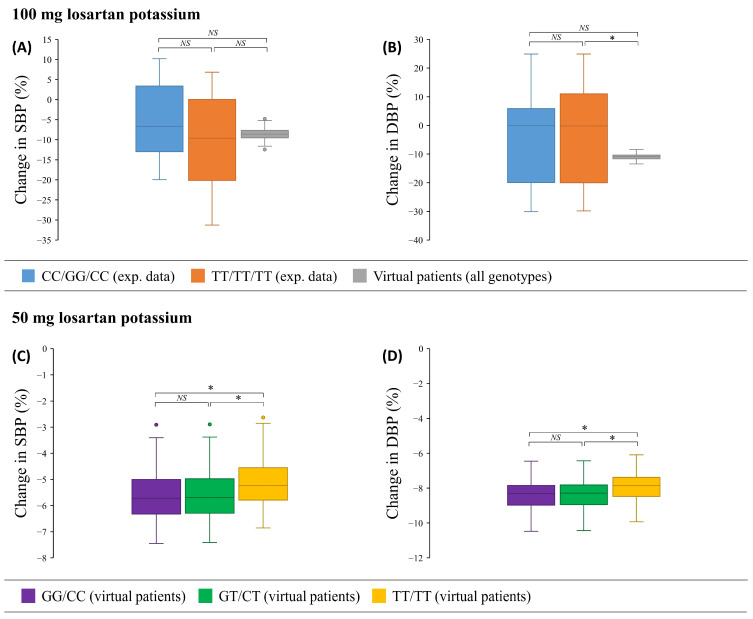
Comparison of simulated and experimentally obtained [53] systolic (**A**) and diastolic (**B**) blood pressure (SBP and DBP, respectively) responses to losartan treatment in real patients with the CC/GG/CC and TT/TT/TT genotypes and in virtual patients (regardless of genotype) using 100 mg oral dose of losartan potassium. Comparison of simulated systolic (**C**) and diastolic (**D**) blood pressure responses to losartan monotherapy in virtual patients with GG/CC, GT/CT, and TT/TT genotypes using 50 mg oral dose of losartan potassium. Box plots show medians, interquartile ranges, maximum, minimum, and outliers. *, *p*-value < 0.0167 (0.05/number of comparison groups, Bonferroni cutoff for significance). *NS*, *p*-value > 0.0167 (0.05/number of comparison groups, Bonferroni cutoff for significance).

Using a single 100 mg oral dose of losartan potassium, the AUC_0-∞, E-3174_ values were similar for all genotypes, and their *k_block_* values did not differ from each other (Supplementary Table S5), suggesting that patients with different *ABCB1* genotypes would respond equally to treatment. To ensure the validity of this result, we compared our simulated blood pressure responses with those obtained clinically [53], assuming that GG/CC and TT/TT genotypes correspond to CC/GG/CC and TT/TT/TT, respectively, as SNPs in the coding region of the *ABCB1* gene are in strong linkage disequilibrium [15,16,17,18,19,20] (Figure 9A,B).

In the experimental case, systolic blood pressure responses for the CC/GG/CC and TT/TT/TT genotypes were not significantly different (*p* = 0.317) and were consistent with those obtained in the virtual patients (CC/GG/CC vs. virtual patients, *p* = 0.085; TT/TT/TT vs. virtual patients, *p* = 0.866, Figure 9A). The diastolic blood pressure responses were also the same for real individuals with CC/GG/CC and TT/TT/TT genotypes (*p* = 0.770), whereas the simulated response differed significantly from the TT/TT/TT group (*p* = 0.010), but not from the CC/GG/CC one (*p* = 0.240, Figure 9B).

In the case of a 50 mg oral dose of losartan potassium, only simulated blood pressure responses in virtual patients with different *ABCB1* genotypes were compared, as we could not find similar experimental data. For both systolic and diastolic blood pressure, the response to losartan treatment was equivalent for the GG/CC and GT/CT genotypes (systolic blood pressure, *p* = 0.760; diastolic blood pressure, *p* = 0.823) and more pronounced than for the TT/TT genotype (*p* < 0.0167, Figure 9C,D).

## 4. Discussion

Genetic factors are known to be able to contribute to an increase in blood pressure by 30–50% [64,65]. Individuals with different *ABCB1* (ATP-binding cassette superfamily, subfamily B, member 1) genotypes may differ significantly in the pharmacokinetics of the antihypertensive drug losartan [41], as well as in its pharmacodynamics (this case was not considered in this paper) [53]. Therefore, the study of *ABCB1* polymorphisms can be employed to personalize drug therapy for arterial hypertension.

The aim of this study was to modify a previously developed model describing the pharmacokinetics of losartan in relation to different *CYP2C9* alleles [36,37] to account for different variants of the *ABCB1* gene. The newly developed model is able to predict the profiles of both losartan and its active metabolite, E-3174, based on the *CYP2C9* and *ABCB1* genotypes of a particular patient simultaneously.

The model showed good agreement with clinical data for both *CYP2C9* and *ABCB1* genotypes; however, a certain discrepancy was observed between the simulated and clinical data for the CYP2C9*3/CYP2C9*3 genotype. The predicted *C*_max, losartan_ was significantly lower than the experimental value (646.686 vs. 1040.908 nM), while the same characteristic for the CYP2C9*1/CYP2C9*1 genotype fell within the mean ± *SD* clinical range (435.059 vs. 555.923 ± 232.679 nM). This divergence can be attributed to the fact that using only one parameter *k_m*, which characterizes the difference between the CYP2C9*1/CYP2C9*1 and CYP2C9*3/CYP2C9*3 genotypes, is not enough to approximate clinical data for both genotypes simultaneously; therefore, further improvement of the model is required. On the one hand, the study by J. Bae and coauthors [52], used for model validation, demonstrated that *C*_max, losartan_ for the CYP2C9*3/CYP2C9*3 genotype was higher than the same variable for CYP2C9*1/CYP2C9*1 (1040.908 vs. 555.923 ± 232.679 nM). On the other hand, Ü. Yasar and colleagues [33] showed that these values were similar for the same genotypes (706 vs. 675 ± 417 nM). This phenomenon is probably caused by ethnic differences: in one study the patients were Korean [52], while in another study [33] Swedish patients were evaluated.

The predicted pharmacokinetic parameters exhibited discrepancies for both the CYP2C9*3/CYP2C9*3 genotype and other genotypes (Figure 6). Clearly, the model underpredicts the value of *t*_1/2_ in all cases. One potential explanation for this phenomenon is that the experimental studies [41,52] did not specify the time points used to calculate this parameter, thereby resulting in the possibility that our points (6, 8, and 10 h) may not correspond to them. It is also noteworthy that, for the purpose of model validation, we used two distinct datasets, each comprising data on a single gene (*CYP2C9* or *ABCB1*). We hypothesized that all patients with unknown *CYP2C9* genotypes were carriers of two CYP2C9*1 alleles, while patients with unknown *ABCB1* genotypes had a GT/CT genotype for this gene. This assumption may not align with actual data, which could also impact the model’s precision.

The primary limitation of this study is related to the small number of patients with CYP2C9*3/CYP2C9*3 genotypes whose clinical data were used for model validation. This is due to the fact that the frequency of this variant allele is also very low (6.3%; https://gnomad.broadinstitute.org/variant/10-94981296-A-C?dataset=gnomad_r4, accessed 5 June 2025). As in the two papers mentioned above [33,52], only one individual with the CYP2C9*3/CYP2C9*3 genotype was used, and, therefore, it was not possible to perform a statistical analysis in this case. Overall, the results obtained should be interpreted with caution, and more data from patients with these genotypes are needed for more accurate model training.

A further limitation of this study pertains to the utilization of two heterogeneous datasets from different investigations [41,52]. While the employment of such data for model training results in more general predictions, it is crucial to acknowledge that different studies may have distinct inclusion criteria and patients from these trials may exhibit divergent physiological characteristics. Consequently, this may cause discrepancies between the model’s predictions and the observed values for a specific group of individuals. This assertion may hold particular relevance for individuals with rare genotypes, such as CYP2C9*3/CYP2C9*3 in our study. A paucity of data has been obtained for these genotypes, and inconsistencies may become particularly evident when values are drawn from disparate studies.

In the future, we intend to refine the model as more experimental data become available not only for specific genotypes for one gene, but also for combinations of two or more genes. In addition, other genes that may influence the absorption, distribution, metabolism, and excretion of losartan can be considered, which will allow more accurate prediction of its pharmacokinetics and pharmacodynamics.

## Figures and Tables

**Table 1:** Characteristics of the main SNPs in the coding region of the human *ABCB1* gene.

rsID	Nucleotide Substitution	Position of the Substitution	Type of Mutation	Amino Acid Substitution
rs1128503	C1236T	13 exon	Synonymous	-
rs2032582	G2677T	22 exon	Nonsynonymous	Ala893Ser
rs2032582	G2677A	22 exon	Nonsynonymous	Ala893Thr
rs1045642	C3435T	27 exon	Synonymous	-

rsID (reference SNP cluster ID), unique number of the single nucleotide polymorphism.

**Table 2:** Characteristics of CYP2C9*2 and CYP2C9*3 alleles.

Allele Name	rsID	Nucleotide Substitution	Position of the Substitution	Type of Mutation	Amino Acid Substitution
CYP2C9*2	rs1799853	C430T	3 exon	Nonsynonymous	Arg144Cys
CYP2C9*3	rs1057910	A1075C	7 exon	Nonsynonymous	Ile359Leu

rsID (reference SNP cluster ID), unique number of the single nucleotide polymorphism.

**Table 3:** Pharmacokinetic parameters of the model and their description.

Pharmacokinetic Parameter	Description
AUC_0–∞_	The area under the concentration–time curve from zero to infinity (nmol·h/L)
AUC_x–y_	AUC from x to y h (nmol·h/L)
*C*_max_	The maximum plasma concentration (nM)
*t*_1/2_	The terminal elimination half-life (h)
*t*_max_	The time at which *C*_max_ occurred (h)
CL/F	The apparent oral clearance of losartan (L/h)

**Table 4:** Fitted values of the E-max model coefficients.

Coefficient	Value
*E*_max_ (unitless)	0.955
*ED*_50_ (nmol·h/L)	5304.326
*α* (unitless)	6.785

**Table 5:** Deviations between experimental datasets for CYP2C9*1/CYP2C9*1 [52] and *ABCB1* genotypes: GG/CC, GT/CT, and TT/TT [41].

*GG/CC* vs. CYP2C9*1/CYP2C9*1	*GT/CT* vs. CYP2C9*1/CYP2C9*1	*TT/TT* vs. CYP2C9*1/CYP2C9*1
322.501	278.345	1510.341

**Table 6:** Comparison of key pharmacokinetic parameters for *CYP2C9* genotypes.

Genotype	Pharmacokinetic Parameter	Clinical Data: Mean ± *SD* (95% Confidence Interval) ^#^	Model Prediction
CYP2C9*1/CYP2C9*1	*C*_max, losartan_ (nM)	555.923 ± 232.679 (408.134, 703.712)	435.059
	*t*_1/2, losartan_ (h)	1.920 ± 0.760 (1.440, 2.400)	1.057 **
	AUC_0–∞, losartan_ (nmol·h/L)	1305.746 ± 241.665 (1152.282, 1459.210)	1352.644
	CL/F (L/h)	94.000 ± 18.000 (82.000, 106.000)	80.182 *
	*C*_max, E-3174_ (nM)	1200.046 ± 192.493 (1077.592, 1322.499)	983.035 **
	*t*_1/2, E-3174_ (h)	4.290 ± 0.400 (4.040, 4.540)	3.360 **
	AUC_0–∞, E-3174_ (nmol·h/L)	7946.670 ± 1067.063 (7268.711, 8624.628)	7708.524
CYP2C9*3/CYP2C9*3	*C*_max, losartan_ (nM)	1040.908	646.686
	*t*_1/2, losartan_ (h)	4.720	1.194
	AUC_0–∞, losartan_ (nmol·h/L)	3156.538	2444.236
	CL/F (L/h)	37.000	44.373
	*C*_max, E-3174_ (nM)	43.717	23.418
	*t*_1/2, E-3174_ (h)	10.560	3.771
	AUC_0–∞, E-3174_ (nmol·h/L)	917.601	196.994

*C*_max_, maximum plasma concentration; *t*_1/2_, terminal elimination half-life; AUC_0–∞_, area under the concentration–time curve from zero to infinity; CL/F, apparent oral clearance of losartan. ^#^, data from [52]. *, the predicted value of the parameter does not fall within the 95% confidence interval, but falls within the mean ± *SD* experimental range. **, the predicted value of the parameter does not fall within the mean ± *SD* experimental range.

**Table 7:** Comparison of key pharmacokinetic parameters for *ABCB1* genotypes.

Genotype	Pharmacokinetic Parameter	Clinical Data ^#^	Model Prediction
GG/CC	*C*_max, losartan_ (nM)	574.1 ± 238.3	350.7
	*t*_max, losartan_ (h)	2.0 (0.5–4.0)	1.6
	*t*_1/2, losartan_ (h)	2.4 ± 0.8	1.5 *
	AUC_0–∞, losartan_ (nmol·h/L)	1649.3 ± 698.2	1349.1
	CL/F (L/h)	83.0 ± 30.6	80.4
	*C*_max, E-3174_ (nM)	1085.8 ± 312.7	885.1
	*t*_max, E-3174_ (h)	4.0 (3.0–6.0)	4.2
	*t*_1/2, E-3174_ (h)	4.7 ± 0.8	4.1
	AUC_0–∞, E-3174_ (nmol·h/L)	7825.9 ± 1560.0	7732.6
	*C*_max, losartan+E-3174_ (nM)	1288.6 ± 334.4	1063.2
GT/CT	*C*_max, losartan_ (nM)	786.0 ± 270.0	435.1 *
	*t*_max, losartan_ (h)	1.5 (0.5–3.0)	1.5
	*t*_1/2, losartan_ (h)	1.9 ± 0.7	1.1 *
	AUC_0–∞, losartan_ (nmol·h/L)	1755.0 ± 436.4	1352.6
	CL/F (L/h)	71.4 ± 19.5	80.2
	*C*_max, E-3174_ (nM)	1192.5 ± 474.0	983.0
	*t*_max, E-3174_ (h)	3.0 (2.0–6.0)	3.8
	*t*_1/2, E-3174_ (h)	4.6 ± 0.9	3.4 *
	AUC_0–∞, E-3174_ (nmol·h/L)	8276.3 ± 1383.3	7708.5
	*C*_max, losartan+E-3174_ (nM)	1445.2 ± 482.7	1192.4
TT/TT	*C*_max, losartan_ (nM)	1033.4 ± 475.5	965.4
	*t*_max, losartan_ (h)	1.0 (0.5–1.5)	0.9
	*t*_1/2, losartan_ (h)	2.2 ± 0.4	0.6 *
	AUC_0–∞, losartan_ (nmol·h/L)	1788.4 ± 506.6	1391.4
	CL/F (L/h)	70.9 ± 18.7	78.0
	*C*_max, E-3174_ (nM)	1484.6 ± 464.6	1207.0
	*t*_max, E-3174_ (h)	2.0 (1.5–4.0)	2.3
	*t*_1/2, E-3174_ (h)	4.6 ± 0.4	2.9 *
	AUC_0–∞, E-3174_ (nmol·h/L)	8614.5 ± 1577.2	7441.6
	*C*_max, losartan+E-3174_ (nM)	1884.0 ± 484.8	1608.8

*C*_max_, maximum plasma concentration; *t*_max_, time at which *C*_max_ occurred; *t*_1/2_, terminal elimination half-life; AUC_0–∞_, area under the concentration–time curve from zero to infinity; CL/F, apparent oral clearance of losartan; ^#^, data from [41]. Values are given as arithmetic mean ± *SD*, except for *t*_max_ which is the median (range). *, the predicted value of the parameter does not fall within the mean ± *SD* experimental range (or the range in the case of *t*_max_).

## Data Availability

The original data presented in the study are openly available in the web version of the BioUML platform in the ABCB1_CYP2C9_losartan_metabolism project (https://sirius-web.org/bioumlweb/#de=data/Collaboration%20(git)/ABCB1_CYP2C9_losartan_metabolism/Data/Diagrams/Final%20model, accessed 5 June 2025). The description of all files in this project and the instructions on how to reproduce our results can be found in the GitHub (https://github.com/DBgentech2023sirius/ABCB1, accessed 5 June 2025) and GitLab (https://gitlab.sirius-web.org/virtual-patient/ABCB1_CYP2C9_losartan_metabolism, accessed 5 June 2025) repositories. Our model is also available in the BioModels database [42] with ID MODEL2504020001 (https://www.ebi.ac.uk/biomodels/MODEL2504020001, accessed 5 June 2025).

## Supplementary Materials

The following supporting information can be downloaded at: https://www.mdpi.com/article/10.3390/pharmaceutics17070935/s1, Table S1: Model reaction equations; Table S2: Parameters and variables of reaction equations; Table S3: Other model equations; Table S4: Parameters and variables of other model equations; Table S5: Correspondence between AUC_0–∞, E-3174_ and *k_block_* values for *ABCB1* genotypes for oral doses of 50 and 100 mg losartan; Table S6: Digitized concentration-time data for *CYP2C9*1/CYP2C9*1* and *CYP2C9*3/CYP2C9*3* genotypes (J. Bae et al., 2011 [52]); Table S7: Digitized concentration-time data for *ABCB1* genotypes: GG/CC, GT/CT, and TT/TT (Shin et al., 2020 [41]); Table S8: Digitized blood pressure responses to losartan treatment (daily oral dose of 100 mg for 6 weeks) for *ABCB1* genotypes CC/GG/CC and TT/TT/TT (Göktaş et al., 2016 [53]); Table S9: Optimized values of the model global parameters; Table S10: Comparison of the AUClosartan+E-3174 (area under the concentration-time curve) of different time periods for *ABCB1* genotypes; Table S11: Median values and standard deviation of each optimized model parameter for all *ABCB1* genotypes (GG/CC, GT/CT, and TT/TT) used to model between-subject variability of losartan and E-3174 plasma curves; Table S12: Sensitivity analysis of the model parameters according to the *ABCB1* genotypes; Figure S1: IC50 plot of losartan; experimental points correspond to oral doses of losartan of 25 mg (point 1), 50 mg (point 2), and 100 mg (point 3); Figure S2: Identifiability plots of the model parameters; Figure S3: Normal distribution of model global parameters; Figure S4: Normal distribution of the parameter k_ent_int, rate constant of reverse transport of losartan from enterocytes to the intestinal lumen by *ABCB1* (h^−1^), for each *ABCB1* genotype; Figure S5: Distribution of physiological characteristics of the population of 100 virtual hypertensive patients.
