# Supplementary material for: Modeling the Influence of CYP2C9 and ABCB1 Gene Polymorphisms on the Pharmacokinetics and Pharmacodynamics of Losartan
Source: Pharmaceutics. 2025 Jul 20;17(7):935. doi: 10.3390/pharmaceutics17070935 (PMC12298228; doi:10.3390/pharmaceutics17070935)
Supplement: Supplementary file 1 [file pharmaceutics-17-00935-s001.zip › pharmaceutics-3669996-supplementary.pdf]

1. Supplementary materials

Table S1. Model reaction equations

The number of reaction in Figure 2	Equation	A physiological process in the body described by the equation
1	$max(0, a*sin(2*\pi/b*time))*[L_{stm}]$	Open-close cycles of the gastric pyloric valve
2	$k\_int\_ent*[L_{int}]$	Absorption of losartan from the intestinal lumen into enterocytes
3	$k\_ent\_int*[L_{ent}]$	Reverse transport of losartan from enterocytes to the intestinal lumen by ABCB1
4	$k\_ent\_cc*[L_{ent}]$	Absorption of losartan from enterocytes into the blood
5	$k_m*[L_{cc}]*start\_CYP2C9$	Conversion of losartan to E-3174 by CYP2C9
6	$k_{elm}*[C_{cc}]$	E-3174 elimination from the body
7	$k_{elp}*[L_{cc}]$	Losartan elimination from the body
8	$k_{12}*[L_{cc}]$	Losartan transfer from the blood to other organs and tissues
9	$k_{21}*[L_{pc}]$	Losartan transfer from organs and tissues into the blood

Table S2. Parameters and variables of reaction equations

Parameter/variable	Definition
a	The amplitude of the sinusoidal equation, which describes open-close cycles of the gastric pyloric valve (h^{-1})
b	The period of the sinusoidal equation, which describes open-close cycles of the gastric pyloric valve (h)
k_int_ent	The rate constant of the losartan absorption from the intestinal lumen into enterocytes (h^{-1})
k_ent_int	The rate constant of reverse transport of losartan from enterocytes to the intestinal lumen by ABCB1 (h^{-1})
k_ent_cc	The rate constant of the losartan absorption from enterocytes into the blood (h^{-1})
k_m	The rate constant of the conversion of losartan to E-3174 by CYP2C9 (h^{-1})
k_{elm}	The E-3174 elimination rate constant (h^{-1})
k_{elp}	The losartan elimination rate constant (h^{-1})
k_{12}	The rate constant of losartan transfer from the blood to other organs and tissues (h^{-1})
k_{21}	The rate constant of losartan transfer from organs and tissues into the blood (h^{-1})
$[L_{stm}]$	The amount of losartan in the stomach (pmol)
$[L_{int}]$	The amount of losartan in the intestine (pmol)
$[L_{ent}]$	The amount of losartan in enterocytes (pmol)
$[L_{cc}]$	The amount of losartan in the blood (pmol)
$[L_{pc}]$	The amount of losartan in organs and tissues (pmol)
$[C_{cc}]$	The amount of E-3174 in the blood (pmol)
$start\_CYP2C9$	The discrete variable used to describe the delay in conversion of losartan to E-3174, which can take the values 0 (no CYP2C9 activity) or 1 (presence of CYP2C9 activity)

Table S3. Other model equations

Equation	Definition
When time > T: *start_CYP2C9 = 1*	The discrete event used to describe the delay in the conversion of losartan to E-3174 for time T
$[L_{stm}](0) = 0.05/461.01*10^{12}$	The initial assignment that determines the 50 mg oral dose of losartan potassium (pmol): 0.05 g = 50 mg, 461.01 g/mol - molecular weight of losartan potassium, 10^{12} - mole to pmole conversion coefficient
$k_{elp} = CL_p/V_{p1}$	The algebraic equation that determines the value of k_{elp}
$k_{elm} = CL_m/V_m$	The algebraic equation that determines the value of k_{elm}
$k_{12} = Q/V_{p1}$	The algebraic equation that determines the value of k_{12}
$k_{21} = Q/V_{p2}$	The algebraic equation that determines the value of k_{21}
$C_p = [L_{cc}]/(V_{p1}*1000)$	The algebraic equation that determines the value of C_p
$C_m = [C_{cc}]/(V_m*1000)$	The algebraic equation that determines the value of C_m
$C_{total} = C_p + C_m$	The algebraic equation that determines the value of C_{total}

Table S4. Parameters and variables of other model equations

Parameter/variable	Definition
T	The delay time of losartan conversion to E-3174 (h)
$[L_{stm}](0)$	The amount of losartan in the stomach at the beginning of the model simulation (pmol)
CL_p	Apparent clearance of losartan (L/h)
CL_m	Apparent clearance of E-3174 (L/h)
V_{p1}	Apparent volume of distribution of losartan in the blood (L)
V_{p2}	Apparent volume of distribution of losartan in organs and tissues (L)
V_m	Apparent volume of distribution of E-3174 in the blood (L)
Q	Apparent clearance of losartan transfer between the blood and other organs and tissues (L/h)
C_p	Concentration of losartan in the blood (nM)
C_m	Concentration of E-3174 in the blood (nM)
C_{total}	Concentration of both losartan and E-3174 in the blood (nM)

Table S5. Correspondence between $AUC_{0-\infty,\ E\text{-}3174}$ and k_{block} values for *ABCB1* genotypes for oral doses of 50 and 100 mg losartan

Genotype	$AUC_{0-\infty,\ E\text{-}3174}$ (nmol·h/L)	k_{block} (unitless)
50 mg losartan		
GG/CC	7732.612	0.886
GT/CT	7708.524	0.885
TT/TT	7441.624	0.867
100 mg losartan		
GG/CC	15465.224	0.954
GT/CT	15417.048	0.954
TT/TT	14883.247	0.954

Table S6. Digitized concentration-time data for *CYP2C9*1/CYP2C9*1* and *CYP2C9*3/CYP2C9*3* genotypes (J. Bae et al., 2011 [52])

CYP2C9*1/CYP2C9*1			***CYP2C9*3/CYP2C9*3***		
**Time (h)**	**Losartan (nM)**	**E-3174 (nM)**	**Time (h)**	**Losartan (nM)**	**E-3174 (nM)**
0.000	0.000	-	0.000	0.146	0.000
0.491	211.385	-	0.481	79.567	0.000
0.984	465.419	30.736	0.992	222.789	0.000
1.491	463.945	247.170	1.500	1039.889	0.000
1.998	287.769	533.110	1.997	621.606	0.000
2.990	191.176	948.322	2.990	355.607	20.840
4.000	130.581	1081.530	4.005	240.408	29.851
5.994	67.541	749.190	5.998	159.353	40.473
7.995	30.933	482.927	8.010	123.250	43.115
10.013	-	339.156	9.990	82.794	42.338
23.997	-	36.657	12.019	77.866	-
-	-	-	23.990	-	15.838

Table S7. Digitized concentration-time data for *ABCB1* genotypes: *GG/CC*, *GT/CT*, and *TT/TT* (Shin et al., 2020 [41])

**GG/CC**			**GT/CT**			**TT/TT**		
**Time (h)**	**Losartan (nM)**	**E-3174 (nM)**	**Time (h)**	**Losartan (nM)**	**E-3174 (nM)**	**Time (h)**	**Losartan (nM)**	**E-3174 (nM)**
0.000	5.338	0.000	0.011	10.676	6.908	0.023	5.338	3.454
0.506	74.733	6.908	0.471	317.616	6.908	0.471	798.043	24.180
1.000	346.975	34.542	0.989	448.399	72.539	1.000	733.986	597.582
1.494	360.320	196.891	1.483	587.189	276.339	1.494	475.089	1129.534
2.000	330.961	431.779	1.989	421.708	614.853	1.989	298.932	1336.788
3.000	269.573	808.290	3.000	226.868	1070.812	3.000	160.142	1222.798
3.989	216.192	987.910	3.977	170.819	1039.724	4.011	112.100	1056.995
6.023	120.107	732.297	6.011	106.762	708.117	6.000	53.381	590.674
8.046	56.050	490.501	8.023	48.043	493.955	8.011	32.028	386.874
10.011	32.028	355.786	10.023	34.698	348.877	10.011	21.352	255.613

Table S8. Digitized blood pressure responses to losartan treatment (daily oral dose of 100 mg for 6 weeks) for *ABCB1* genotypes CC/GG/CC and TT/TT/TT (Göktaş et al., 2016 [53]).

CC/GG/CC		TT/TT/TT	
**Changes in SBP, %**	**Changes in DBP, %**	**Changes in SBP, %**	**Changes in DBP, %**
10.208	24.928	6.848	24.928
8.096	12.560	6.656	12.560
6.848	6.248	0.032	11.143
-0.064	5.475	-0.160	11.014
-6.208	0.193	0.032	0.064
-6.688	-0.064	-6.592	0.064
-7.552	0.064	-6.688	-0.322
-7.072	-9.984	-12.640	-0.193
-6.400	-15.652	-13.408	-0.064
-13.408	-20.032	-12.544	-9.984
-14.176	-20.161	-20.224	-9.984
-12.544	-19.903	-21.376	-20.161
-19.936	-30.081	-20.128	-20.032
-	-	-31.264	-20.032
-	-	-	-29.823

SBP, systolic blood pressure; DBP, diastolic blood pressure.

Table S9. Optimized values of the model global parameters

Model parameter	Initial value	Fitted value
CL_m	16.456	6.374
CL_p	30.401	43.859
T	0.819	0.500
Vm	57.598	26.192
Vp_1	56.175	13.173
Vp_2	135.630	33.890
Q	217.103	134.072
a	6.645	3.685
*k_a**	5.127	-
*k_ent_cc***	-	6.418
*k_int_ent***	-	12.663

*, the parameter is absent in the current model; **, the parameter is absent in the initial model (Babaev et al., 2024, 2025 [36, 37]).

Table S10. Comparison of the $AUC_{losartan+E\text{-}3174}$ (area under the concentration-time curve) of different time periods for *ABCB1* genotypes

Genotype	Time range (h)	Clinical data: mean ± *SD* (nmol·h/L)#	Model prediction (nmol·h/L)
GG/CC	0 - 2	687.4 ± 472.0	846.2
	2 - 4	2039.3 ± 831.4	2038.0
	4 - 6	1990.4 ± 491.7	1933.4
	6 - 8	1338.2 ± 321.4	1466.8
	8 - 10	903.6 ± 190.9	1010.0
	10 - ∞	2516.5 ± 663.3	1787.4*
	0 - 4	2726.7 ± 1235.3	2884.1
	0 - 6	4717.1 ± 1445.1	4817.6
	0 - 8	6055.2 ± 1507.8	6284.4
	0 - 10	6958.8 ± 1570.8	7294.4
	0 - ∞	9475.3 ± 1923.5	9081.8
GT/CT	0 - 2	1088.5 ± 601.4	1084.2
	2 - 4	2390.0 ± 848.9	2323.2
	4 - 6	1967.8 ± 613.2	1963.9
	6 - 8	1303.1 ± 379.9	1364.7
	8 - 10	867.3 ± 253.9	881.0
	10 - ∞	2414.5 ± 811.7	1444.2*
	0 - 4	3478.6 ± 1052.4	3407.4
	0 - 6	5446.3 ± 1330.0	5371.3
	0 - 8	6749.5 ± 1446.0	6736.0
	0 - 10	7616.8 ± 1496.2	7617.0
	0 - ∞	10031.5 ± 1561.8	9061.2
TT/TT	0 - 2	2286.0 ± 958.7	2273.4
	2 - 4	2778.4 ± 577.1	2448.3
	4 - 6	1784.0 ± 485.4	1570.4
	6 - 8	1058.2 ± 268.4	978.2
	8 - 10	682.7 ± 164.1	602.1
	10 - ∞	1813.7 ± 488.7	960.7*
	0 - 4	5064.4 ± 1185.6	4721.7
	0 - 6	6848.4 ± 1289.5	6292.0
	0 - 8	7906.5 ± 1364.8	7270.2
	0 - 10	8589.3 ± 1414.1	7872.3
	0 - ∞	10403.0 ± 1683.1	8833.1

#, data from (Shin et al., 2020 [41]).

*, the predicted value of the parameter does not fall within the mean ± *SD* experimental range.

Table S11. Median values and standard deviation of each optimized model parameter for all *ABCB1* genotypes (GG/CC, GT/CT, and TT/TT) used to model between-subject variability of losartan and E-3174 plasma curves

Model parameter	Median value			10 % *SD*		
	GG/CC	GT/CT	TT/TT	GG/CC	GT/CT	TT/TT
CL_m	6.374			0.637		
CL_p	43.859			4.386		
T	0.500			0.050		
Vm	26.192			2.619		
Vp_1	13.173			1.317		
Vp_2	33.890			3.389		
Q	134.072			13.407		
a	3.685			0.369		
k_ent_cc	6.418			0.642		
k_int_ent	12.663			1.266		
k_ent_int	151.485	101.800	$1.431*10^{-12}$	15.149	10.180	$1.431*10^{-13}$

Table S12. Sensitivity analysis of the model parameters according to the *ABCB1* genotypes.

Genotype	Variable	Parameter												
		a	*b*	*k_int_ent*	*k_ent_int*	*k_ent_cc*	*k_m*	*T*	*CL_m*	*Vm*	*CL_p*	*Vp_1*	*Q*	*Vp_2*
GG/CC	$AUC_{0-\infty, losartan}$	6.1E-03	-5.8E-03	6.3E-03	-5.8E-03	6.5E-03	-4.5E-01	2.3E-02	-1.8E-08	-2.3E-07	-5.4E-01	-4.5E-01	-1.9E-03	-8.7E-04
	$AUC_{0-\infty, E\text{-}3174}$	-7.3E-03	7.0E-03	-7.6E-03	7.0E-03	-7.9E-03	5.4E-01	-2.7E-02	-1.0E+00	1.1E-07	-5.5E-01	5.5E-01	2.3E-03	1.0E-03
	$C_{max, losartan}$	8.9E-02	-6.0E-03	6.0E-01	-5.8E-01	6.3E-01	-3.4E-01	2.0E-02	3.9E-08	4.2E-08	-4.1E-01	-4.0E-01	3.3E-02	-2.2E-01
	$C_{max, E\text{-}3174}$	4.5E-02	4.3E-02	3.1E-01	-3.0E-01	3.3E-01	5.7E-01	-2.0E-02	-4.3E-01	-5.7E-01	-5.2E-01	5.6E-01	1.7E-02	-6.2E-02
GT/CT	$AUC_{0-\infty, losartan}$	8.3E-03	-7.9E-03	8.2E-03	-7.2E-03	8.6E-03	-4.5E-01	3.1E-02	2.7E-08	5.4E-07	-5.4E-01	-4.5E-01	-2.6E-03	-1.2E-03
	$AUC_{0-\infty, E\text{-}3174}$	-1.0E-02	9.6E-03	-9.8E-03	8.7E-03	-1.0E-02	5.4E-01	-3.7E-02	-1.0E+00	-2.5E-08	-5.5E-01	5.5E-01	3.2E-03	1.4E-03
	$C_{max, losartan}$	1.1E-01	-2.1E-02	5.3E-01	-5.0E-01	5.6E-01	-3.3E-01	2.7E-02	-4.2E-09	5.5E-08	-4.0E-01	-3.9E-01	2.7E-02	-2.4E-01
	$C_{max, E\text{-}3174}$	4.5E-02	4.6E-02	2.4E-01	-2.3E-01	2.5E-01	5.8E-01	-2.9E-02	-3.9E-01	-6.1E-01	-5.1E-01	5.7E-01	2.2E-02	-8.0E-02
TT/TT	$AUC_{0-\infty, losartan}$	3.2E-02	-3.1E-02	1.4E-02	-1.6E-15	2.1E-02	-4.2E-01	1.2E-01	1.7E-07	1.0E-06	-5.5E-01	-4.4E-01	-1.0E-02	-4.5E-03
	$AUC_{0-\infty, E\text{-}3174}$	-4.1E-02	3.9E-02	-1.8E-02	2.0E-15	-2.6E-02	5.4E-01	-1.5E-01	-1.0E+00	-8.6E-08	-5.8E-01	5.6E-01	1.3E-02	5.8E-03
	$C_{max, losartan}$	3.2E-01	-2.0E-01	4.3E-02	-2.0E-14	1.3E-01	-2.4E-01	1.0E-01	-6.3E-08	-2.2E-07	-3.2E-01	-3.5E-01	-5.6E-02	-2.8E-01
	$C_{max, E\text{-}3174}$	2.2E-02	6.6E-02	-1.5E-02	3.5E-16	-1.6E-02	6.2E-01	-1.4E-01	-2.5E-01	-7.5E-01	-4.9E-01	6.0E-01	5.2E-02	-1.7E-01

a - the amplitude of the sinusoidal equation, which describes open-close cycles of the gastric pyloric valve (h^{-1}); *b* - the period of the sinusoidal equation, which describes open-close cycles of the gastric pyloric valve (h); *k_int_ent* - rate constant of the losartan absorption from the intestinal lumen into enterocytes (h^{-1}); *k_ent_int* - rate constant of reverse transport of losartan from enterocytes to the intestinal lumen by ABCB1 (h^{-1}); *k_ent_cc* - rate constant of the losartan absorption from enterocytes into the blood (h^{-1}); *k_m* - rate constant of the conversion of losartan to E-3174 by CYP2C9 (h^{-1}); *T* - time delay in the conversion of losartan to E-3174 (h); *CL_m* - apparent clearance of E-3174 (L/h); *Vm* - apparent volume of distribution of E-3174 in the blood (L); *CL_p* - apparent clearance of losartan (L/h); *Vp_1* - apparent volume of distribution of losartan in the blood (L); *Q* - apparent clearance of losartan transfer between the blood and other organs and tissues (L/h); *Vp_2* - apparent volume of distribution of losartan in other organs and tissues (L).
$AUC_{0-\infty}$ - area under the concentration-time curve from zero to infinity (nmol·h/L); C_{max} - maximum plasma concentration (nM).

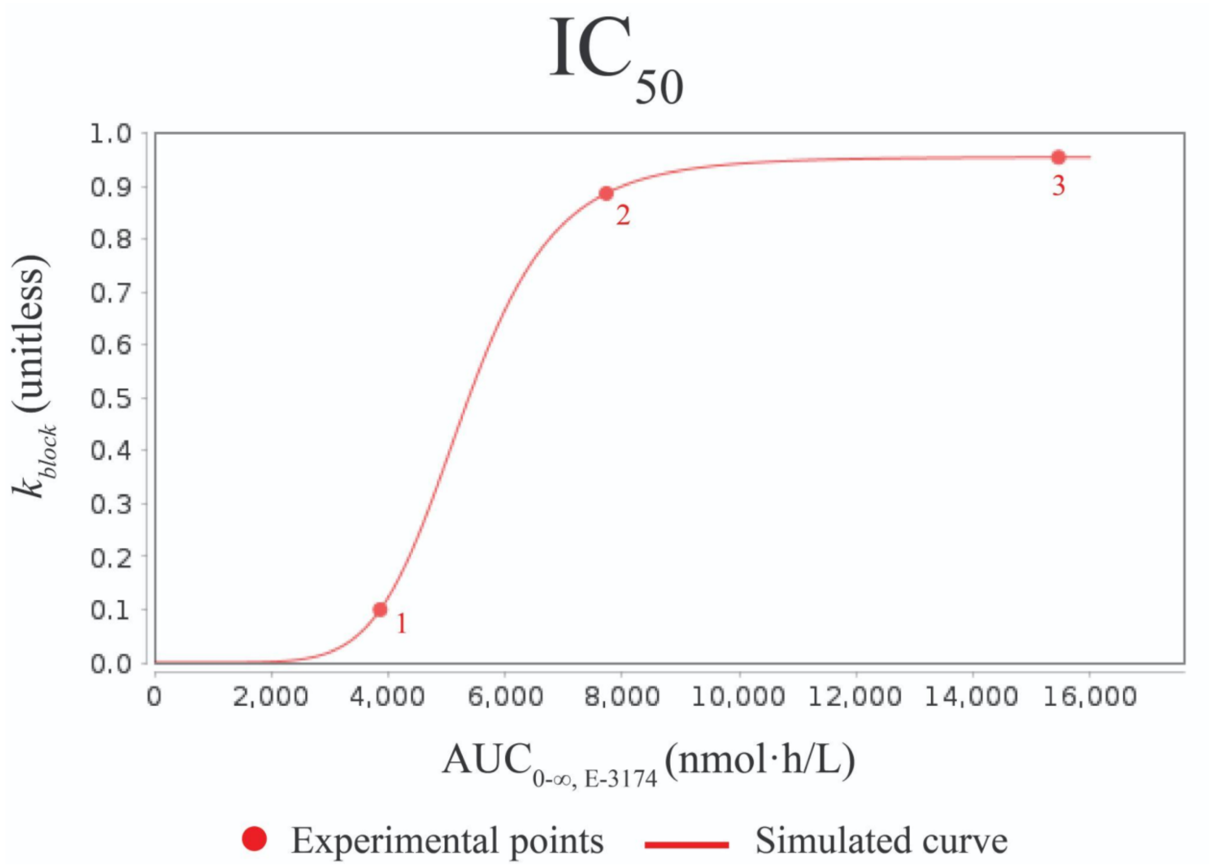

Figure. S1. IC50 plot of losartan; experimental points correspond to oral doses of losartan of 25 mg (point 1), 50 mg (point 2), and 100 mg (point 3).

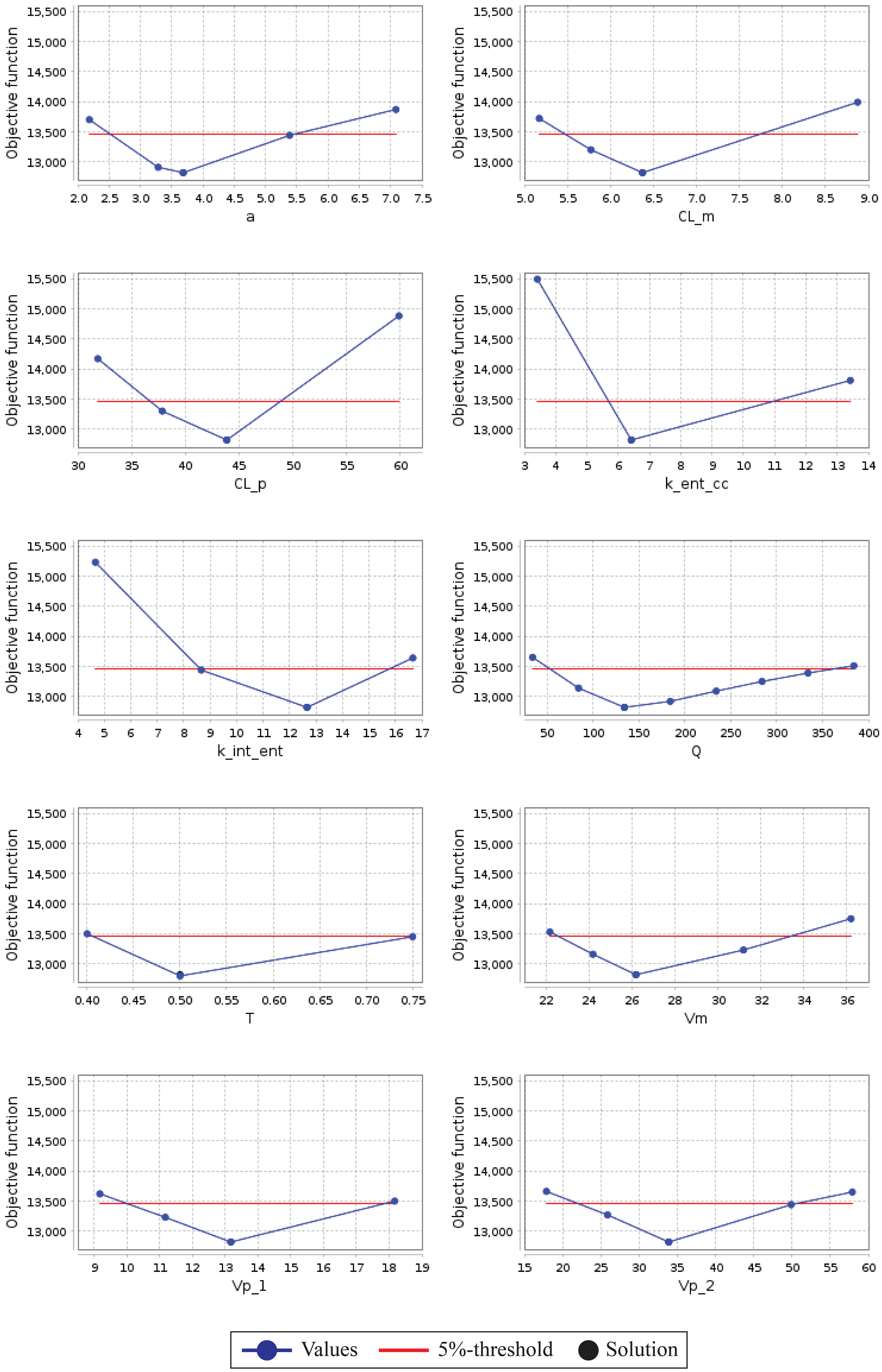
Objective function
15,500
15,000
14,500
14,000
13,500
13,000
a
CL_m
CL_p
k_ent_cc
k_int_ent
Q
T
Vm
Vp_1
Vp_2
Values
5%-threshold
Solution

Figure. S2. Identifiability plots of the model parameters: a - the amplitude of the sinusoidal equation, which describes open-close cycles of the gastric pyloric valve (h^{-1}); CL_m - apparent clearance of E-3174 (L/h); CL_p - apparent clearance of losartan (L/h); k_ent_cc - rate constant of the losartan absorption from enterocytes into the blood (h^{-1}); k_int_ent - rate constant of the losartan absorption from the intestinal lumen into enterocytes (h^{-1}); Q - apparent clearance of losartan transfer between the blood and other organs and tissues (L/h); T - time delay in the conversion of losartan to E-3174 (h); Vm - apparent volume of distribution of E-3174 in the blood (L); Vp_1 - apparent volume of distribution of losartan in the blood (L); Vp_2 - apparent volume of distribution of losartan in other organs and tissues (L). The blue dots represent the objective function values, the black dot indicates the optimal solution (coincides with the objective function value at the zero step of optimization), and the red line denotes the boundary value of the objective function (5 % of the initial objective function value).

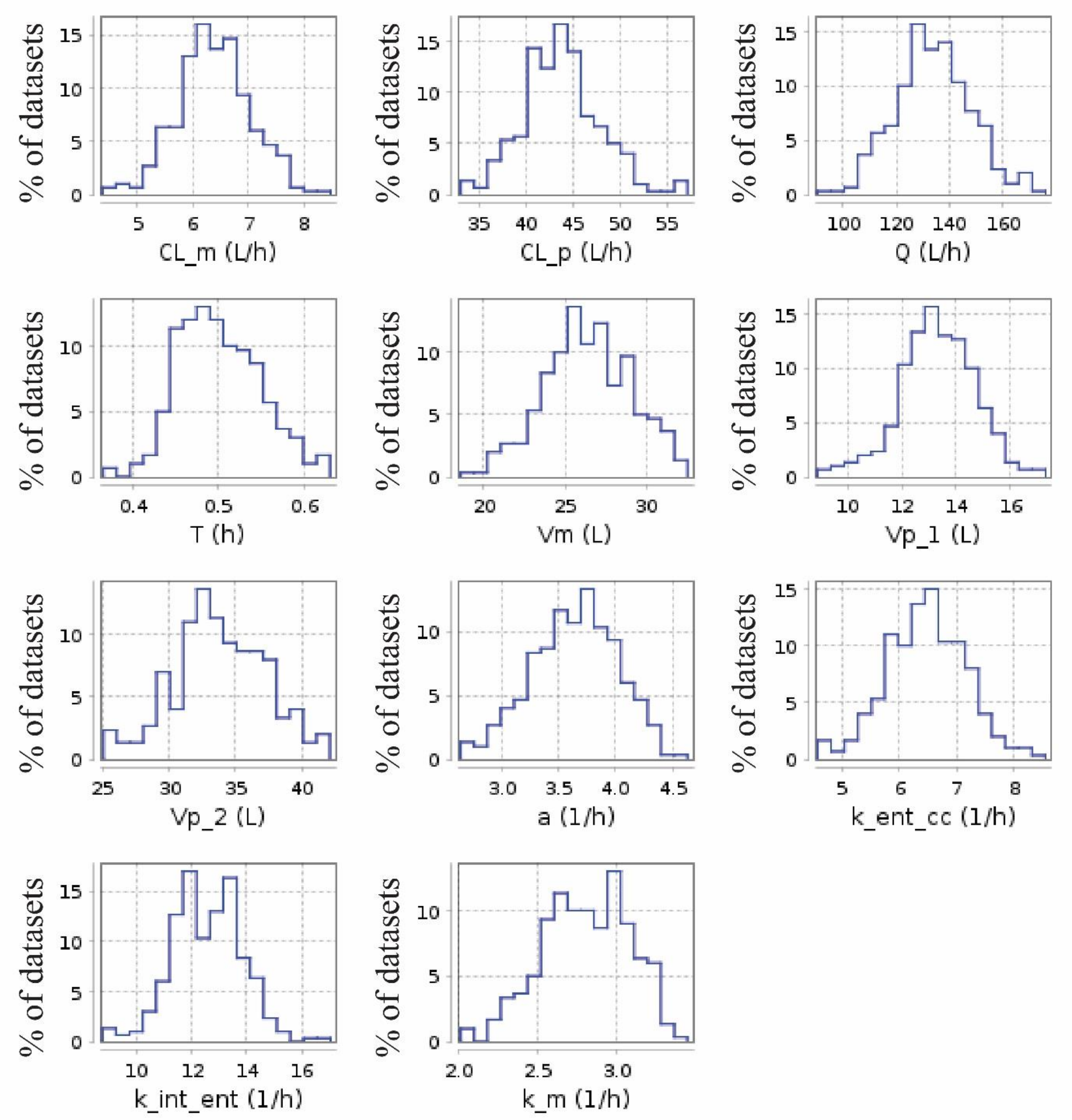

Figure S3. Normal distribution of model global parameters: CL_m - apparent clearance of E-3174 (L/h); CL_p - apparent clearance of losartan (L/h); Q - apparent clearance of losartan transfer between the blood and other organs and tissues (L/h); T - time delay in the conversion of losartan to E-3174 (h); Vm - apparent volume of distribution of E-3174 in the blood (L); Vp_1 - apparent volume of distribution of losartan in the blood (L); Vp_2 - apparent volume of distribution of losartan in other organs and tissues (L); a - the amplitude of the sinusoidal equation, which describes open-close cycles of the gastric pyloric valve (h^{-1}); k_ent_cc - rate constant of the losartan absorption from enterocytes into the blood (h^{-1}); k_int_ent - rate constant of the losartan absorption from the intestinal lumen into enterocytes (h^{-1}); k_m - rate constant of the conversion of losartan to E-3174 by CYP2C9 (h^{-1}).

All distributions were generated with 300 values (100 values for each of the three *ABCB1* genotypes).

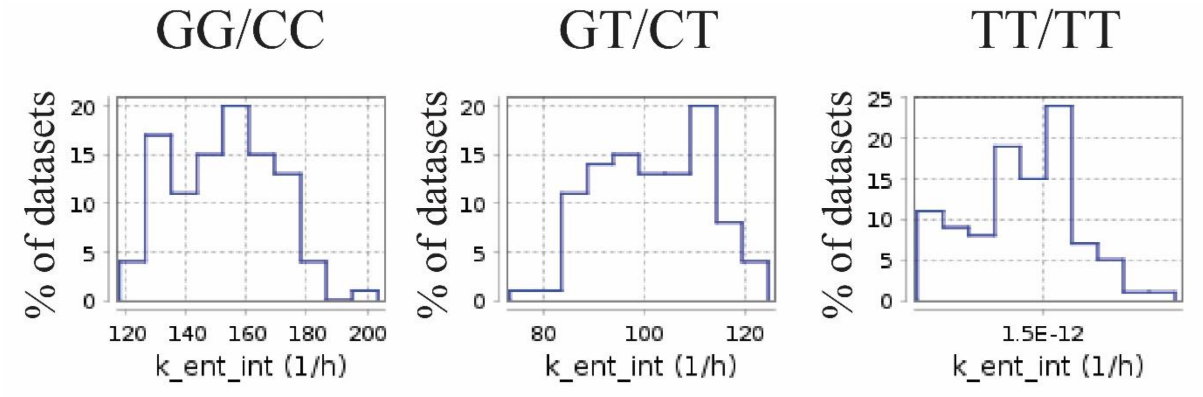

Figure S4. Normal distribution of the parameter k_ent_int, rate constant of reverse transport of losartan from enterocytes to the intestinal lumen by ABCB1 (h^{-1}), for each *ABCB1* genotype.

All distributions were generated with 100 values.

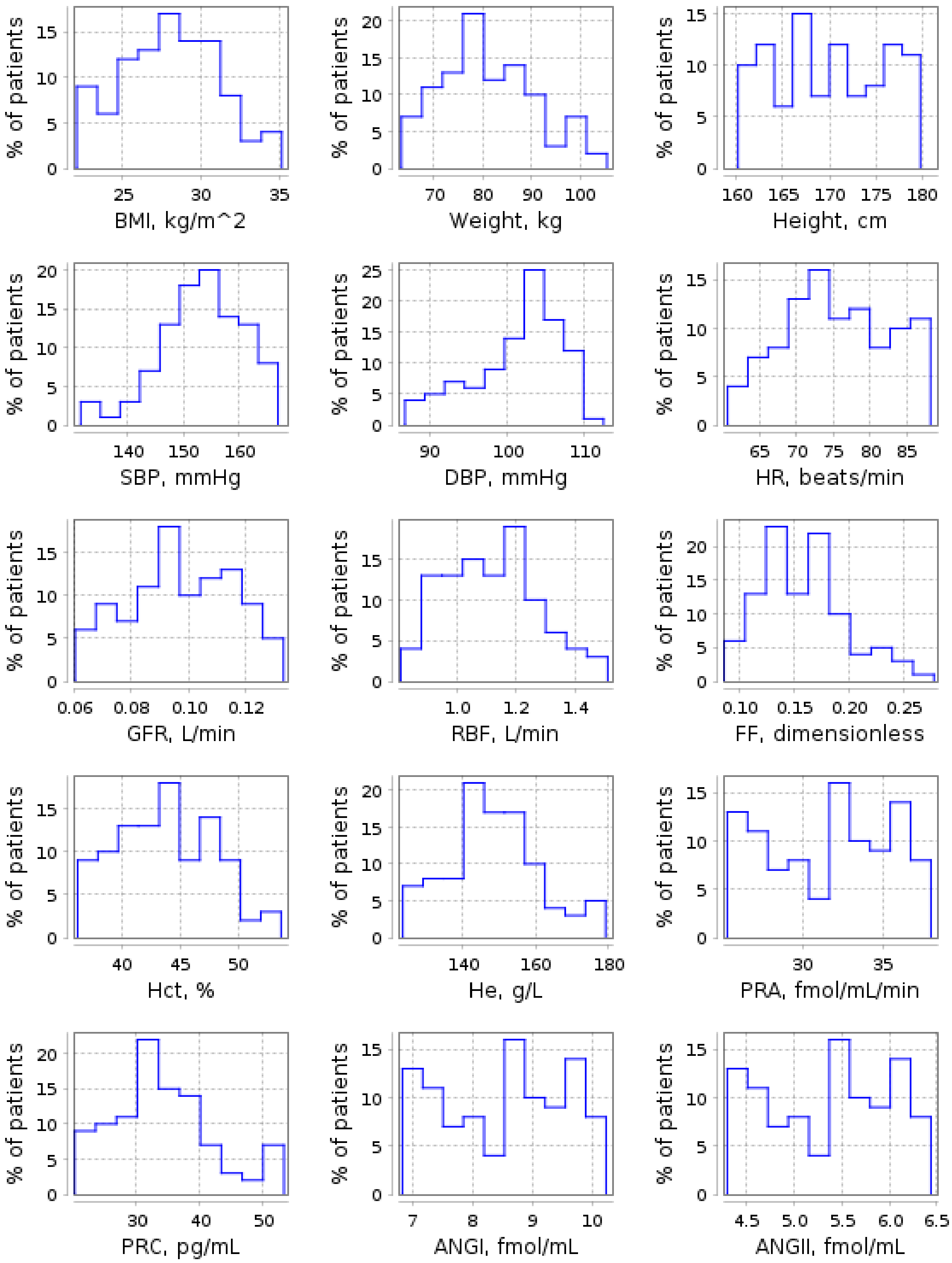

Figure S5. Distribution of physiological characteristics of the population of 100 virtual hypertensive patients. BMI, body mass index; SBP, systolic blood pressure; DBP, diastolic blood pressure; HR, heart rate; GFR, glomerular filtration rate; RBF, renal blood flow; FF, filtration fraction; Hct, hematocrit; He, hemoglobin; PRA, plasma renin activity; PRC, plasma renin concentration; ANGI, plasma angiotensin I concentration; ANGII, plasma angiotensin II concentration.
